# NF90–NF45 is essential for β cell compensation under obesity-inducing metabolic stress through suppression of p53 signaling pathway

**DOI:** 10.1038/s41598-022-12600-y

**Published:** 2022-05-25

**Authors:** Sylvia Lai, Takuma Higuchi, Masayuki Tsuda, Yasunori Sugiyama, Keiko Morisawa, Taketoshi Taniguchi, Shuji Sakamoto

**Affiliations:** 1 Laboratory of Molecular Biology, Science Research Center, Kochi Medical School, Kochi University, Oko-cho, Kohasu, Nankoku, Kochi 783-8505 Japan; 2 Division of Laboratory Animal Science, Science Research Center, Kochi Medical School, Kochi, Japan; 3 Department of Life Sciences, Faculty of Agriculture, Kagawa University, Kagawa, Japan

**Keywords:** Cell biology, Molecular biology

## Abstract

The Nuclear Factor 90 (NF90)–NF45 complex has been known to regulate the progression of transcription, mRNA stability, translational inhibition, RNA export and microRNA biogenesis. However, the physiological functions of the NF90–NF45 complex remain unclear. We newly discovered that the NF90–NF45 complex was expressed in primary β cells and established cell lines. Therefore, in this study, we focused on the function of the endogenous NF90–NF45 complex in the β cells. To investigate this issue, we generated β-cell-specific NF90–NF45 deficient mice. These mice exhibited hyperglycaemia and lower plasma insulin levels under a high fat diet together with decreased islet mass. To uncover this mechanism, we performed a whole-genome expression microarray of the total RNA prepared from β cell lines treated with siRNAs targeting both NF90 and NF45. In this result, we found an activation of p53 signaling in the NF90–NF45-knockdown cells. This activation was supported by elevation of luciferase activity derived from a reporter plasmid harboring p53 binding sites in the NF90–NF45-knockdown cells. Furthermore, the knockdown of NF90–NF45 resulted in a significant retardation of the β cell line growth rates. Importantly, a dominant negative form of p53 rescues the growth retardation in BTC6 cells depleted of NF90–NF45, suggesting that NF90–NF45 would be positively involved in β cell proliferation through suppression of p53 signal pathway. Taken together, NF90–NF45 is essential for β cell compensation under obesity-inducing metabolic stress via repression of p53 signaling.

## Introduction

Nuclear Factor 90 (NF90), encoded by Interleukin Enhancer Binding Factor 3 (ILF3), (also referred as NFAR1 or DRBP76), is a double-stranded RNA binding protein. NF90 contains a nuclear localization signal, two double-stranded RNA binding motifs, a zinc finger nucleic acid-binding domain and a glutamic acid-rich region. NF90 forms a complex with a distinct protein, Nuclear Factor 45 (NF45, also referred as Interleukin Enhancer Binding 2, ILF2), which has various biological functions including transcription^1–3^, mRNA stability^4^, translational inhibition^5^ and RNA export^6^. We previously demonstrated that the NF90–NF45 complex suppresses microRNA (miRNA) biogenesis through binding to primary-miRNAs, which are transcribed from miRNA genes^7,8^. These NF90–NF45 functions were found by experiments in vitro. However, the function of NF90–NF45 in vivo remains unclear. To elucidate the role of NF90 and the NF90–NF45 complex in vivo, we previously generated and studied NF90 and NF45 transgenic (Tg) mice. NF90 Tg mice showed skeletal muscular atrophy and heart failure owing to mitochondria degeneration^9^, while the NF90–NF45 double Tg mice showed a decrease in muscular mass caused by the development of centronuclear myopathy through the suppression of myogenic miRNA biogenesis^10^. These observations showed that the overexpression of NF90 or NF90–NF45 causes pathophysiological events by the alteration of RNA metabolism (including miRNA biogenesis). However, the physiological functions of endogenous NF90 and NF45 are largely unknown because the NF90- or NF45-deficient mice exhibited perinatal lethality owing to respiratory failure or embryonic lethality^1,11^.

In this study, we attempted to unravel the function of the endogenous NF90–NF45 complex. Here, we found that the endogenous NF90 family-NF45 complex is expressed robustly in pancreatic islet cells. To investigate the role of the endogenous NF90–NF45 in the islet cells, we generated β-cell-specific NF90 and NF45-deficient mice (β NF45−/− mice). Of note, we found that the mice developed hyperglycaemia because of a reduction in the plasma insulin level under a high fat diet (HFD)-feeding. It was thought that the decreased plasma insulin level was caused by the smaller islet size in the β NF45−/− mice compared with that in the control mice under the HFD-feeding. Intriguingly, phosphorylation of Histone H3 (Ser10) (pH3), which is a marker of cell proliferation, was apparently observed in islets of control mice fed with HFD, whereas the phosphorylation was significantly reduced in islets of β NF45−/− mice under HFD-feeding. Therefore, these observations imply that the down-regulation of NF90–NF45 causes smaller islets by growth retardation in β cells under the obesity-inducing metabolic stress. Furthermore, depletion of NF90 and NF45 in the pancreatic β cell lines showed cell growth inhibition by activation of p53 signaling which is known to participate in cell cycle arrest. Collectively, these findings suggest that NF90–NF45 would be associated with the pancreatic β cell compensation under diabetic condition through cell proliferation promoted by the arrest of the p53 signal pathway.

## Results

### NF90–NF45 is highly expressed in murine pancreatic β cells

**Figure 1:**
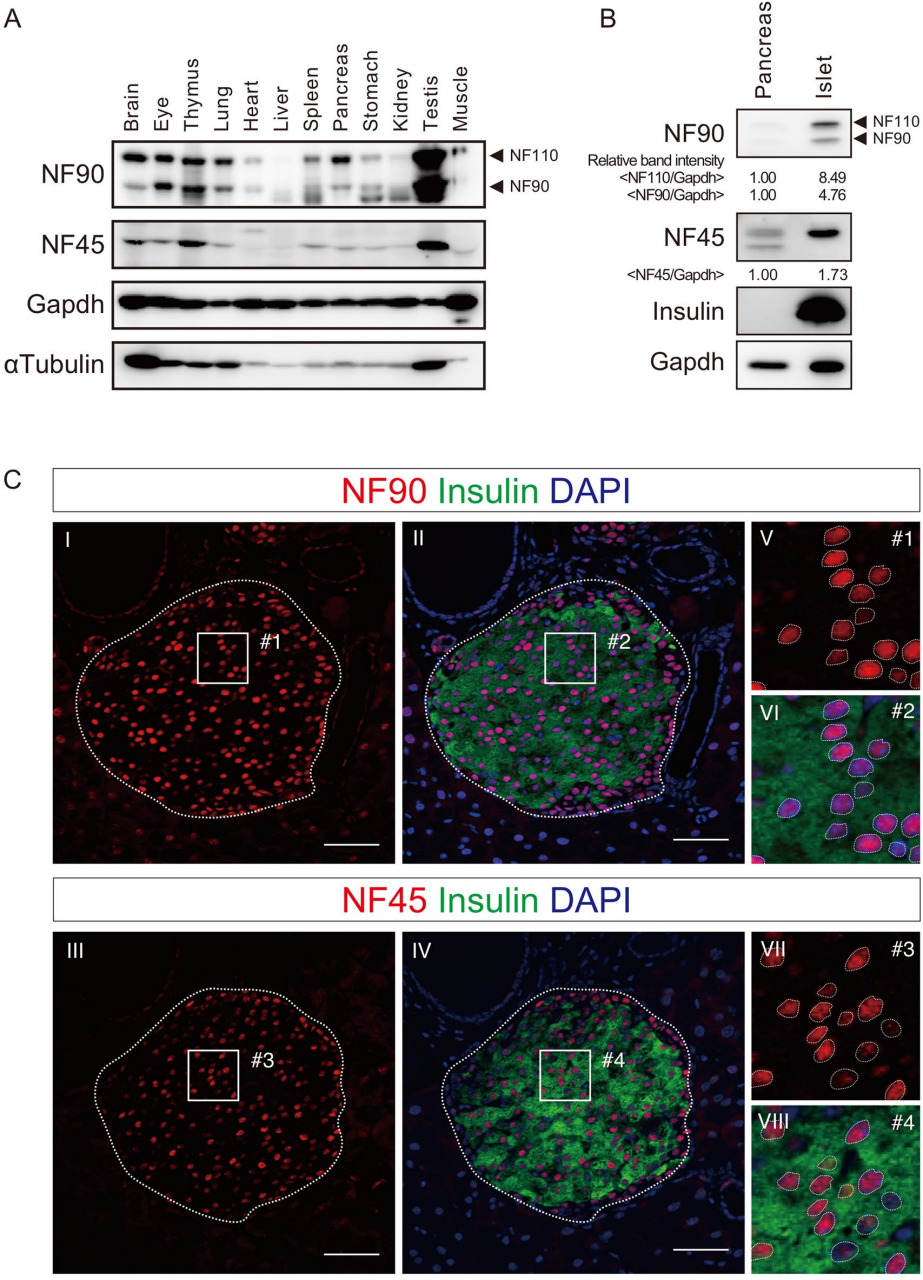
NF90 family and NF45 is expressed in pancreatic β cells. (**A**) Expression levels of NF90 family and NF45 were examined in mice tissues by western blot. Gapdh and α Tubulin was used as an internal control. Experiment is representative of n = 3 mice tissues. (**B**) Western blot analysis of levels of NF90 family and NF45 in pancreas or isolated islet from wild-type mice. Insulin and Gapdh was used as an islet marker and an internal control, respectively. Intensities of specific bands was measured by densitometry. Gapdh was used for normalization of data. Experiment is representative of n = 3 mice pancreas and n = 6 mice islets. (**C**) Immunofluorescence staining of NF90 family (red, panels I, II, V and VI) and NF45 (red, panels III, IV, VII and VIII) in pancreas section of wild-type mice. Insulin (Green) and DAPI (Blue) was shown as β cells and nuclei, respectively. Bar, 50 µm. In panels I to IV, an islet was shown as a dash-line. Higher magnifications of #1 to #4 in panels I to IV were presented in panels V to VIII. In panels V to VIII, a nucleus was shown as a dash-line.

To determine the physiological function of the NF90–NF45 complex in normal tissues, we first examined the expression of the NF90 family member composed of NF90 and NF110, and NF45 in murine tissues by western blot. As previously reported, the NF90 family and NF45 were highly expressed in the brain, thymus and testis (Fig. 1A)^11,12^. The expression of these proteins also was observed in the eye, lung, spleen, stomach and kidney, whereas the heart, liver and skeletal muscle exhibited only a slight expression (Fig. 1A). In this analysis, we newly discovered the expression of the NF90 family and NF45 in the pancreas (Fig. 1A). In particular, the expression of NF110, a longer form of the NF90 family, was higher than those of NF90–NF45 in the pancreas (Fig. 1A). In turn, we carried out western blot analysis using both whole pancreas and isolated islets from the pancreas. As a result, the NF90 and NF45 as well as NF110 exhibited higher expression at the protein level in the islets than the whole pancreas (Fig. 1B). To assess the localization of the NF90 family and NF45 in the pancreas, we performed immunohistochemistry in the pancreas section of mice. This analysis revealed that the NF90 family and NF45 were robustly expressed in the pancreatic β cells (Fig. 1C, panels I–IV). Moreover, the subcellular localization of the NF90 family and NF45 was predominantly in the nucleus of the β cells (Fig. 1C, panels V–VIII).

### β cells specific NF90–NF45 deficient mice develop Type 2 diabetes symptoms

**Figure 2:**
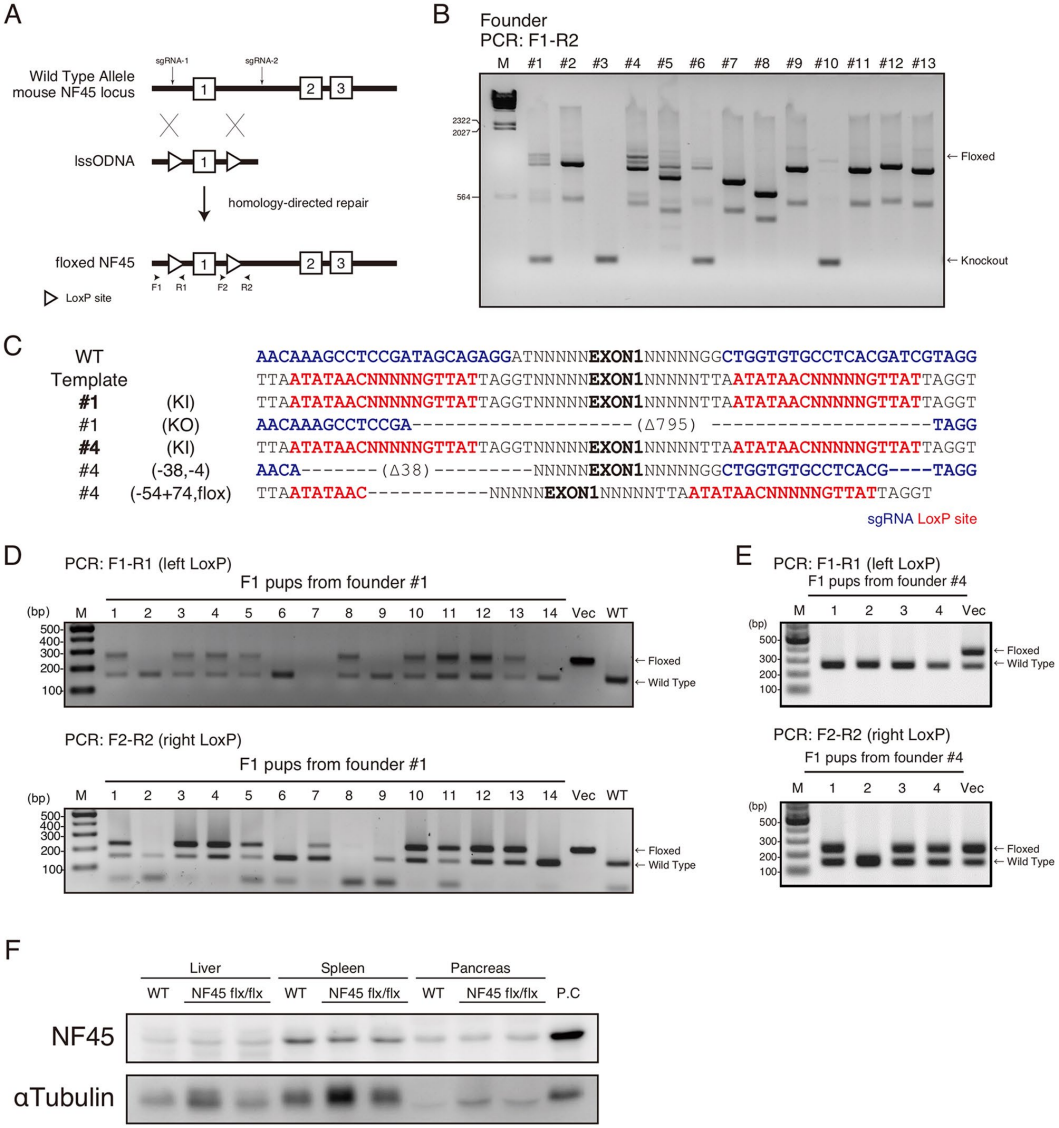
Generation of NF45 flox/flox mice. (**A**) Targeting strategy for the generation of NF45 floxed mice using CRISPR/Cas9 system with two single guide RNAs (sgRNAs) targeted to sequences flanking *NF45* exon 1 (sgRNA-1 and 2) and a long single-stranded oligonucleotide DNA (lssODNA) containing two loxP sites flanked by short arms with homology to the desired insertion sites. (**B**) Genotyping of founder mice (#1–#13) injected with sgRNAs-1 and -2, lssODNA and Cas9 mRNA. PCR products corresponding to floxed NF45 locus or knockout of the NF45 locus were indicated by an arrow. M, λHindIII marker (**C**) DNA sequencing of the CRISPR/Cas9-targeted NF45 locus of founder mice #1 and #4. SgRNAs and loxP sites were showed as blue and red, respectively. (**D** and **E**) Genotyping of F1 pups derived from founder #1 and #4 by PCR using primer pairs targeted loxP sites shown in A. M, 100 bp DNA ladder. (**F**) Western blot analysis of the levels of NF45 in liver, spleen and pancreas from wild-type and NF45 flox/flox (flx/flx) mice. P.C, positive control. Protein extraction from murine testis was used as positive control.

Pancreatic β cells are known to secrete insulin to regulate blood glucose and β cell dysfunction causes Type 2 diabetes. Therefore, we initially attempted to generate mice with a targeted NF45 disruption in the pancreatic β cells (β NF45−/− mice) to ascertain whether the NF90–NF45 downregulation in the pancreatic β cells is correlated with the occurrence of Type 2 diabetes. The β NF45−/− mice were also assumed to exhibit NF90 depletion in the pancreatic β cells because NF90 and NF45 are known to stabilize each other’s protein level^13^. To obtain the β NF45−/− mice, we generated mice with conditional null alleles of the *NF45* gene using CRISPR/Cas9-mediated homology-directed repair (HDR) to insert loxP sites flanking *NF45* exon 1. Mouse zygotes were injected with two single guide RNAs (sgRNAs) targeted to sequences flanking *NF45* exon 1 (sgRNA-1 and 2) (Fig. 2A), and a long single-stranded oligonucleotide DNA (lssODNA) containing two loxP sites flanked by short arms with homology to the desired insertion sites (Fig. 2A) together with Cas9 protein. As a result, we obtained two founder mice (#1 and #4) carrying the two loxP sites flanking *NF45* exon 1 by PCR analysis using genomic DNA from tail tips, followed by DNA sequencing (Fig. 2B,C). The founder mice (#1 and #4) were crossed with wild type mice, and their offspring were genotyped by PCR to determine the existence of a loxP site. We determined that founder #1 has the ability to transmit both loxP sites to the next generation (Fig. 2D), while founder #4 was capable of transmitting only one loxP site to its offspring (Fig. 2E). Therefore, we used the founder #1 line as NF45 flox/flox mice for further analysis. Furthermore, the loxP sites flanking *NF45* exon 1 loci did not affect the NF45 protein expression level (Fig. 2F).

**Figure 3:**
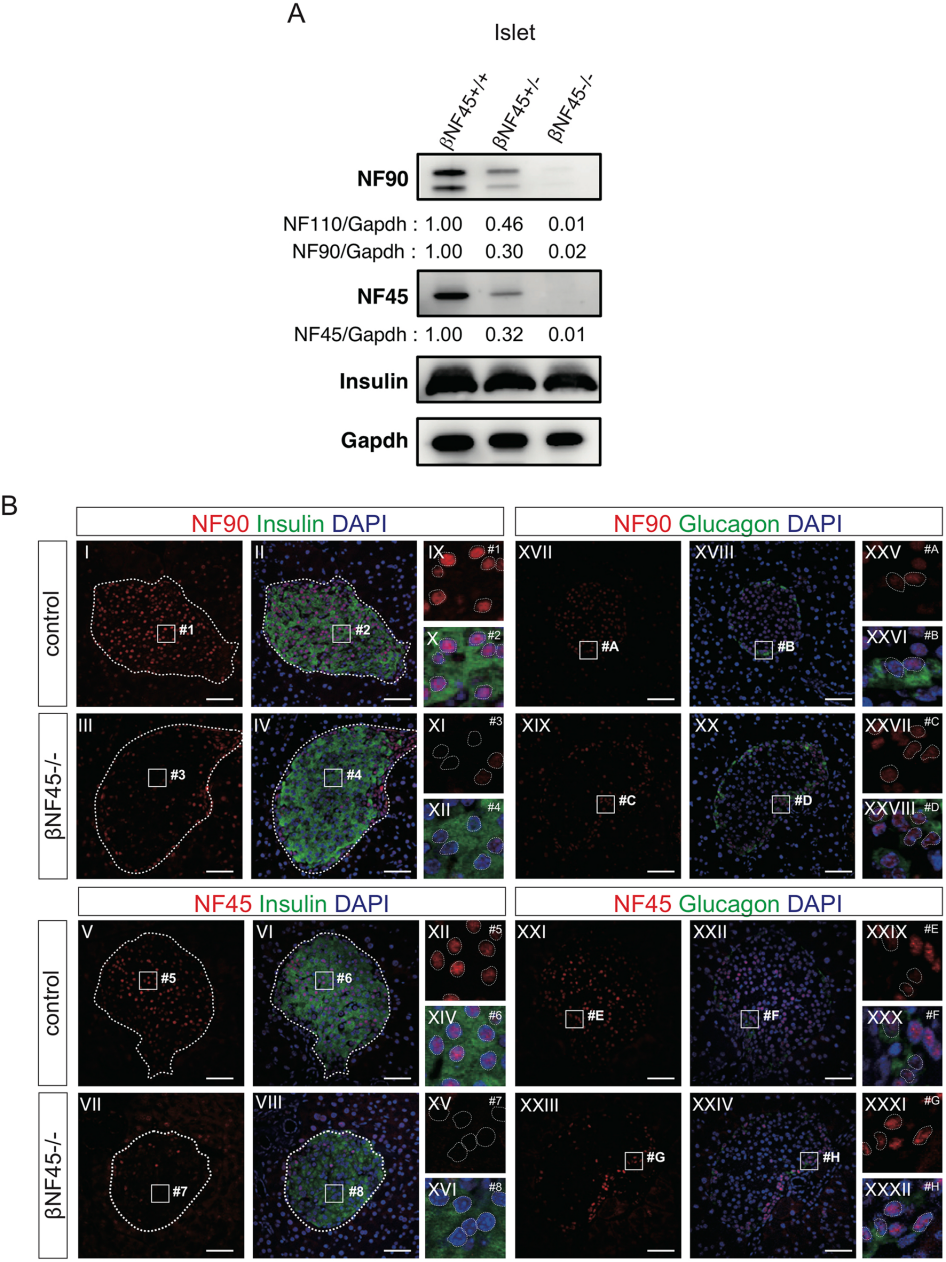

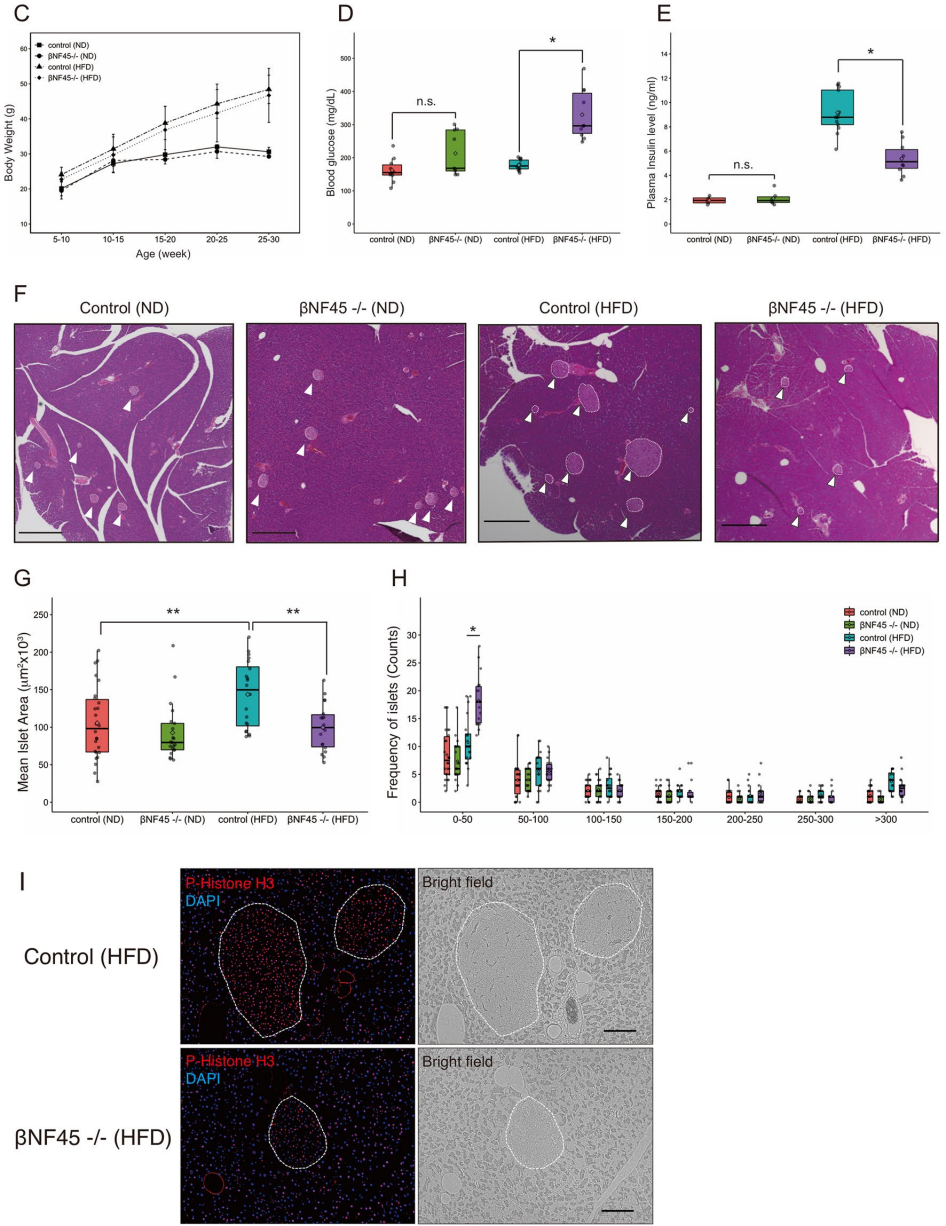

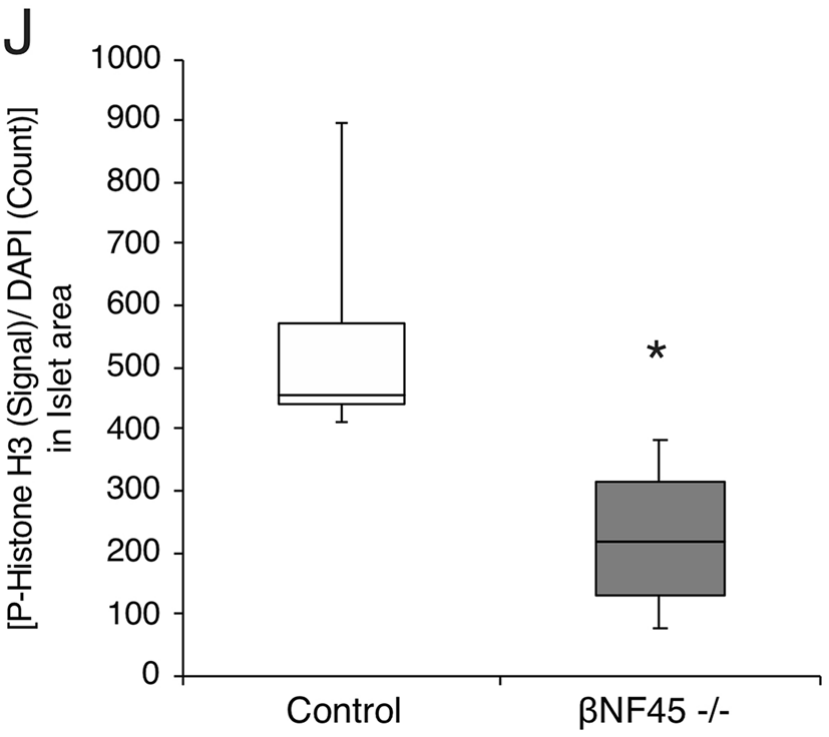
βNF45−/− mice develops Type 2 diabetes symptoms. (**A**) Western blot analysis of the protein level of NF90 and NF45 in islets isolated from control and βNF45−/− mice. α Tubulin was used as an internal control and for normalization of data. Experiment is representative of n = 4 mice islets. (**B**) Immunofluorescence staining of NF90 (red), NF45 (red), Insulin (green) and DAPI (blue) in pancreas section of control and βNF45−/− mice. Bar, 50 µm. An islet was shown as a dash-line. (**C**) Growth curve of control mice (n = 6) and βNF45−/− (n = 5) mice fed with either ND or HFD at 6–28 weeks of age. Data are expressed as means ± standard deviations and are representative of two independent experiments. (**D**) Measurement of the amount of blood glucose of ND-feeding control (n = 4) and βNF45−/− mice (n = 6) and HFD-feeding control (n = 13) and βNF45−/− mice (n = 10) at 28 weeks of age. Data were expressed as means ± standard deviations. *P < 0.05 relative to control by a two-tailed Student’s t test. The data included the result from two independent experiments. (**E**) Measurement of plasma insulin level of ND-feeding control (n = 11) and βNF45−/− mice (n = 16) and HFD-feeding control (n = 13) and βNF45−/− mice (n = 10). Data were expressed as means ± standard deviations. *P < 0.05 relative to control by a two-tailed Student’s t test. The data included the result from two independent experiments. (**F**) H & E staining of pancreas section of either ND-feeding or HFD-feeding control and βNF45−/− mice at 28 weeks of age. Bar, 1 mm. (**G**) Islets were manually traced, and the areas were measured as islet area. More than 400 islets from each group were analysed. The total islet areas were divided by the total number of islets examined and the values were presented as dot plots. **P < 0.01 relative to control by a two-tailed Student’s t test. (**H**) islet size distribution analysis of ND-feeding control (n = 5) and βNF45−/− mice (n = 3) and HFD-feeding control (n = 3) and βNF45−/− mice (n = 3) at 28 weeks of age. The values were presented as dot plots. **P < 0.01 relative to control by a two-tailed Student’s t test. (**I**) Immunofluorescence staining of P-Histone H3 (red) and DAPI (blue) and bright-field image in pancreas section of control (HFD) and βNF45−/− mice (HFD). Bar, 100 µm. An islet was shown as a dash-line. (**J**) Signal intensity of P-Histone H3 in islets of control (n = 4) and βNF45−/− mice (n = 4). The intensities were normalized by the number of DAPI in the islet. The values were presented as box plots. *P < 0.05 relative to control by a two-tailed Student’s t test.

The NF45 flox/flox mice were next crossed with Ins1-Cre transgenic mice^14^; hereafter, the selective β cell deficient NF45 mice were referred as β NF45−/− mice. As mentioned above, NF45 and NF90 were known as binding partners that stabilize each other at the protein level^13^. As expected, the β NF45−/− mice showed reduced NF90 and NF45 expression level in the islet (Fig. 3A). Immunofluorescence staining also showed a defect of NF90 and NF45 in the β cells of the β NF45−/− mice (Fig. 3B, panels XI and XV). However, NF90 and NF45 expression was not altered in other islet cells (Fig. 3B, panels XXV–XXXII) or the exocrine cells (Fig. 3B) in the pancreas. To ascertain the role of NF90–NF45 in the β cells, we initially analyzed the phenotypes in the β NF45−/− mice under normal diet (ND) feeding. However, there was no significant difference in body weight gain, blood glucose concentration and plasma insulin level between control mice (bNF45 ± mice) and the β NF45−/− mice (Fig. 3C–E). Moreover, morphometric analysis by H & E staining indicates that the size of pancreatic islets in β NF45−/− mice was same as that in control mice (NF45 flox and βNF45 ± mice) (Fig. 3G,H, orange and green bars). In turn, to study the glucose metabolism in the β NF45−/− mice under diabetic condition, we fed β NF45−/− male mice with a high fat diet (60% kcal from fat). β NF45+/− mice were used as control under this condition. The body weight gain showed no significant difference between the two groups under the HFD feeding (Fig. 3C). However, the β NF45−/− mice developed hyperglycaemia owing to the fall of insulin level in plasma compared with the control mice (Fig. 3D,E). To explore the cause for the reduced plasma insulin level in the β NF45−/− mice, we carried out the morphometric analysis of the islets from the control mice and the β NF45−/− mice under the HFD feeding. Expectedly, the islets were significantly expanded in the control mice fed with HFD compared with those fed with ND (Fig. 3G,H, comparison of orange and blue bars). Under this obesity-inducing metabolic stress, the β NF45−/− mice showed decreased islet mass (Fig. 3F,G, comparison of blue and purple bars), and exhibited smaller islets by islet size distribution analysis (Fig. 3H, asterisk, comparison of blue and purple bars). These findings imply that an increment in the insulin secretion by the islet expansion in the βNF45 ± mice prevents hyperglycaemia under the stimulation with HFD (see Fig. 3D, lane control (HFD)). Importantly, NF90–NF45 would function as a positive regulator in the expansion of the islets under the diabetic condition because the knockout of NF90–NF45 suppresses the islets expansion under the obesity-inducing metabolic stress (Fig. 3F–H, compared compare lanes control (HFD) and βNF45−/− (HFD)). To investigate whether the fall in islet mass by the down-regulation of NF90–NF45 under the HFD feeding condition is due to β cell proliferation, we analyzed phosphorylation of Histone H3 (Ser10) (pH3), which is a marker of cell proliferation, in islets of the control mice and the β NF45−/− mice fed with HFD by immunohistochemistry. The signal of pH3 was apparently observed in the islets of control mice fed with HFD (Fig. 3I,J). On the other hand, the signal was significantly reduced in the islets of βNF45−/− mice (Fig. 3I,J), suggesting that the decreased islet mass in the islets of βNF45−/− mice would be caused by growth retardation in β cells.

### Depletion of NF90–NF45 activates p53 signaling in pancreatic β cells

**Figure 4:**
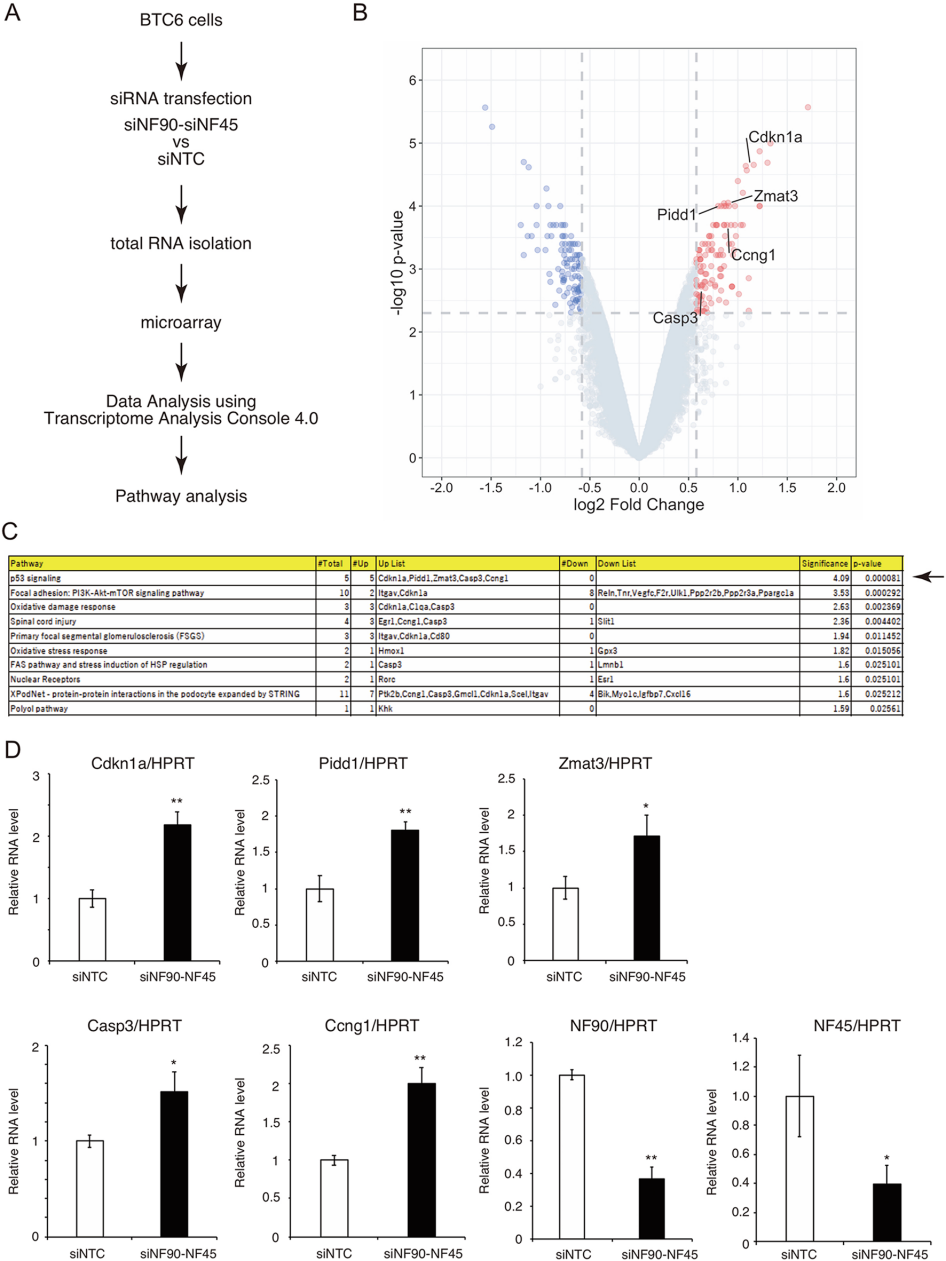
Pathway analysis in β cell lines depleted of NF90 and NF45. (**A**) Schema of an experimental procedure for microarray analysis. Total RNA was prepared from BTC6 cells treated with siNF90 and siNF45 for 7 days. (**B**) Volcano plots with ‘log2 Fold Change’ on the x-axis and ‘− log10 p-value’ on the y-axis reveal significant changes of genes that exhibited significantly different expression levels between siNTC-treated cells and siNF90–NF45-treated cells (values of p < 0.005, fold change of NF90–NF45-treated cells is > 1.5 and < − 1.5, respectively). > 1.5-fold-change, p < 0.005 and < − 1.5-fold-change, p < 0.005 were indicated as red and blue colours, respectively. (**C**) Wikipathway analysis using genes pathed the filter criteria mentioned above (> 1.5 or < − 1.5-fold change, P < 0.005) shown in (**A**). Top 10 pathways were shown as list in order of significance. An arrow indicates top-1 rank by this analysis. (**D**) qRT-PCR of 5-upragurated genes (Cdkn1a, Pidd1, Zmat3, Casp3 and Ccng3) in p53 signaling shown in (**C**) in siNTC- and siNF90–NF45-treated cells. The levels of NF90 and NF45 expression were also measured by qRT-PCR. HPRT was used as an internal control. Data are expressed as mean ± standard deviations. (n = 3 per group). *P < 0.05, **P < 0.01 relative to the siNTC-treated cells by a two-tailed Student’s t test.

**Figure 5:**
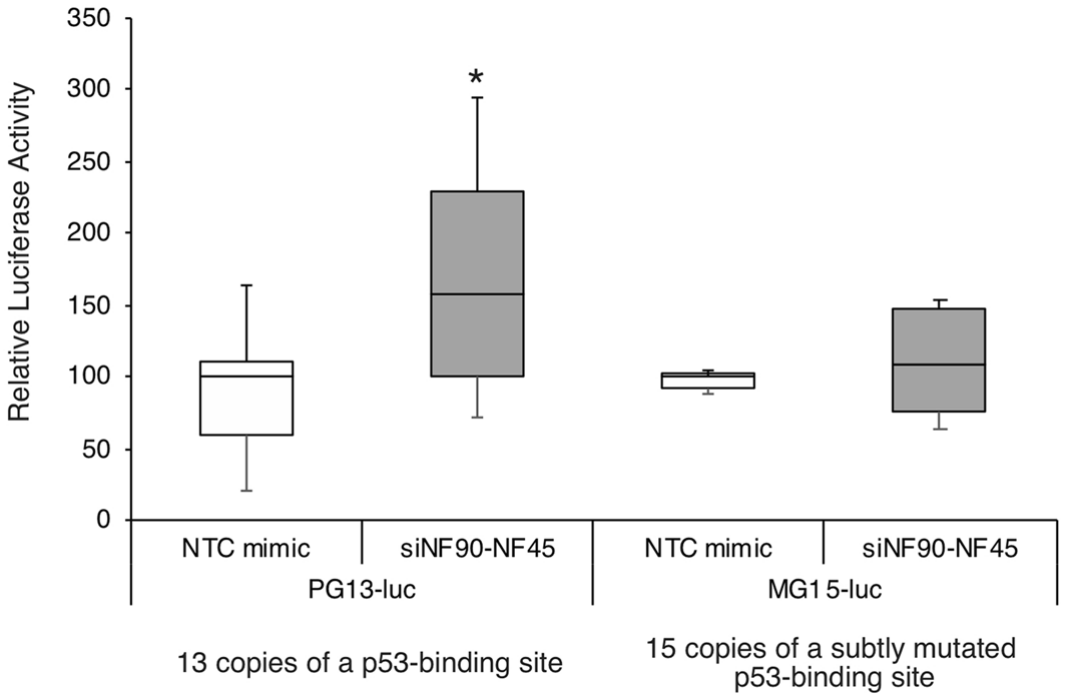
Analysis of p53 signaling in β cell lines depleted of NF90 and NF45. PG13-luc (wt p53 binding sites) and MG15-luc (mut p53 binding sites) were transfected into BTC6 cells treated with either siNTC or siNF90–NF45. Luciferase activities were determined 72 h after transfection. The firefly luciferase activity was normalized to the results of the renilla luciferase activity. Data are expressed as the mean ± standard deviations. (n = 6 per group). *P < 0.05 relative to the siNTC-treated cells by a two-tailed Student’s t test.

To explore NF90–NF45-targeted genes controlling cell growth in β cells, we performed a whole-genome expression microarray of the total RNA prepared from murine pancreatic β cell lines (BTC6 cells) treated with siRNAs targeting both NF90 and NF45 for 7 days (Fig. 4A). As shown in Fig. 4A, the siRNAs targeting NF90 and NF45 suppressed the expression of both proteins. Compared with cells transfected with the non-targeting control siRNA (siNTC), the microarray analysis demonstrated that the NF90–NF45-knockdown cells shared 109 upregulated genes and 103 downregulated genes at > 1.5 or < − 1.5-fold change, P < 0.005 (Fig. 4B and a GEO accession number GSE184853). Continuously, Wikipathways analysis was carried out using the 212 genes pathed the filter criteria (> 1.5 or < − 1.5-fold change, P < 0.005) by Transcriptome Analysis Console (TAC) software (Thermo Fisher Scientific). As a result, top 10 pathways were listed in order of significance shown in Fig. 4C. Among the pathways, we focused on p53 signaling because this pathway is engaged in cell growth, besides ranking as top-1 by this pathway analysis (Fig. 4C). To confirm the reliability of the analysis, we measured the RNA levels of 5 upregulated genes (cdkn1a also termed p21, Pidd1, Zmat3, Casp3 and Ccng3) related to the p53 signaling and NF90–NF45 in BTC6 cells treated with siRNAs targeting NF90–NF45. Expectedly, all of genes were significantly elevated in the NF90–NF45-knockdown cells, while the expression of NF90–NF45 was remarkably decreased in the cells (Fig. 4D). We also measured the level of the 5 up-regulated genes in primary islets from βNF45−/− mice with or without HFD feeding. However, Pidd1 was undetectable in the islets from control mice and βNF45−/− mice with or without HFD feeding. On the other hand, other genes were detected in the primary islets (Supplementary Fig. S1). As a result, there was no significant difference in the expression of all tested genes in the islets between the control mice and the βNF45−/− mice fed with ND (Supplementary Fig. S1). Meanwhile, the expressions of Cdkn1a (p21) and Ccng1 were significantly elevated in the islets of βNF45−/− mice fed with HFD compared with that of control mice (shown as asterisks in Supplementary Fig. S1). In addition, Zmat3 and Casp3 tend to be elevated in the islet of βNF45−/− mice compared with control mice under HFD feeding condition (Supplementary Fig. S1). Therefore, these findings are largely consistent with the phenomenon which is found in the murine pancreatic β cell line (BTC6 cells) depleted with NF90–NF45 (Fig. 4D), suggesting that the p53 signaling in pancreatic β cells is activated by the down-regulation of NF90–NF45 under diabetic condition in vivo. To ascertain activation of the p53 signaling in the BTC6 cells depleted of NF90–NF45 and the primary islet cells of βNF45−/− mice fed with HFD, we utilized PG13-luc and MG15-luc which are luciferase reporter plasmids harboring 13 copies of a p53-binding site and 15 copies of a subtly mutated p53-binding site, respectively^15^. The reporter plasmids were transfected into BTC6 cells treated with either siNTC or siNF90–NF45, and luciferase activity was measured. The result shows that knockdown of NF90–NF45 caused a significant elevation of the luciferase activity in BTC6 cells transfected with PG13-Luc, whereas the activity was not altered by the NF90–NF45 downregulation in the cells transfected with MG15-Luc (Fig. 5). Consistent with the result shown in Fig. 4, this finding implied that the p53 signaling was activated in pancreatic β cell lines when a fall in NF90–NF45 expression.

### The growth retardation of β cells by depletion of NF90–NF45 is correlated with the activation of p53 signaling

**Figure 6:**
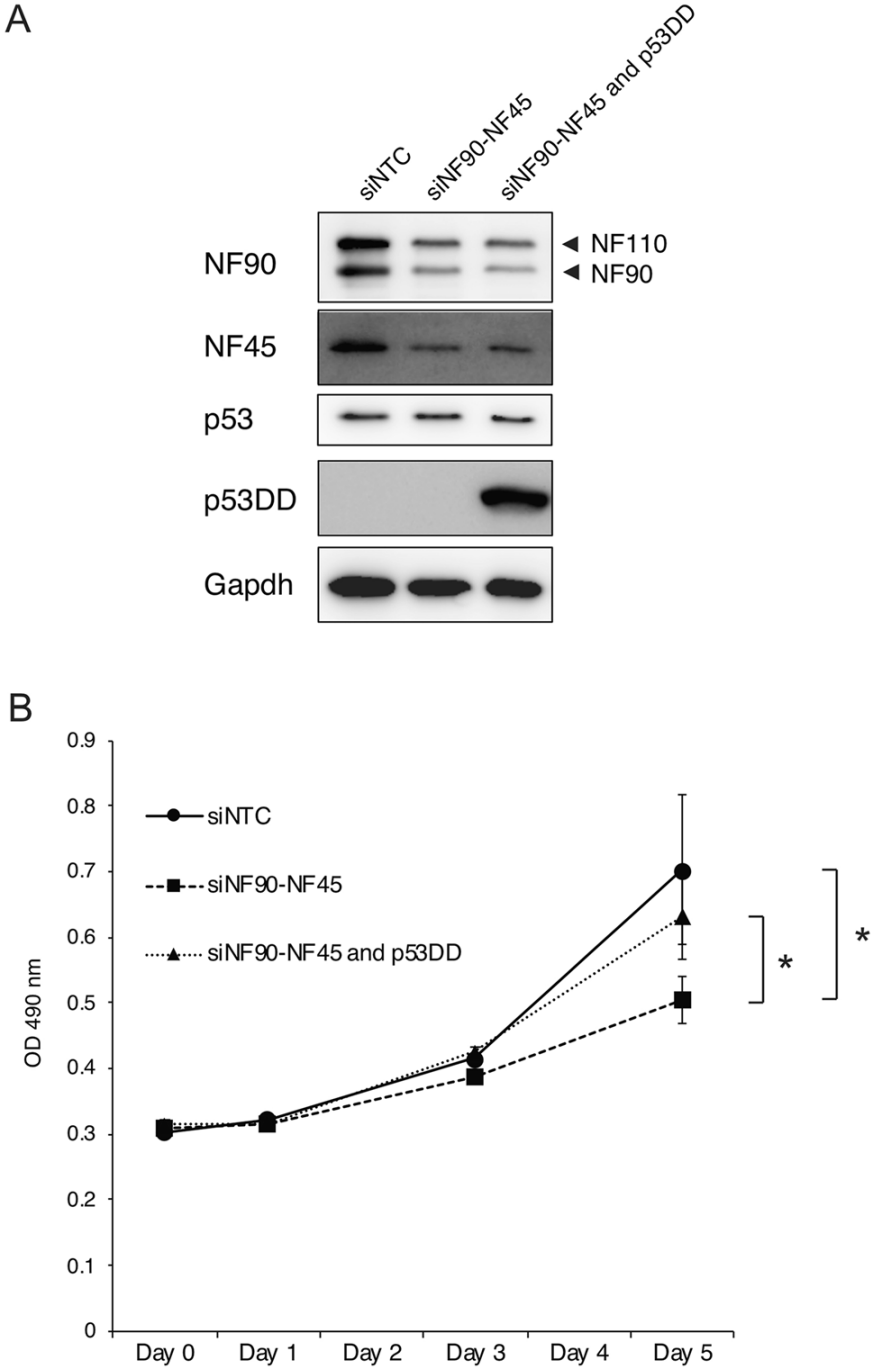
Dominant negative form of p53 (p53DD) recovers the growth retardation by the knockdown of NF90–NF45 in β cell lines. (**A**) BTC6 cells were co-transfected with the indicated siRNA with or without p53DD expression vector. Expression level of NF90, NF45, Gapdh, p53 and p53DD were detected by western blot. Gapdh was used as an internal control. (**B**) Proliferation of BTC6 cells co-transfected with indicated siRNA with or without p53DD expression vector were analyzed by MTS assay. Data were expressed as mean ± standard deviations. (n = 3 per group). *P < 0.05 relative to either siNTC or siNF90–NF45 and p53DD-treated cells by a two-tailed Student’s t test.

To confirm the findings that NF90–NF45 deficiency causes an islet mass decrease in vivo through growth retardation of β cells (Fig. 3F–J), we performed a cell proliferation assay using pancreatic β cell lines deleted of endogenous NF90–NF45 in vitro. We used BTC6 cells depleted of NF90–NF45 by transfection with siRNAs targeting NF90 and NF45. The effect of the siRNAs was verified by western blot. The result showed that the NF90 and NF45 levels were markedly reduced in cells transfected with the siRNAs targeting NF90 and NF45 (Fig. 6A). We found that the NF90–NF45 knockdown caused a significant inhibition of cell proliferation in BTC6 cells by MTS assay (Fig. 6B), suggesting that NF90–NF45 functions as a positive regulator in the cell proliferation of pancreatic β cells.

Furthermore, together with the observation shown in Figs. 4 and 5, the growth retardation of β cells by depletion of NF90–NF45 would be correlated with the activation of p53 signaling. To confirm this, we performed cell viability assay using pCE-mp53DD (a dominant negative form of p53) which is known to inhibit p53 function^16^. As shown in Fig. 6B, co-transfection of pCE-mp53DD with siNF90–NF45 rescues the growth retardation in BTC6 cells. These data imply that the reduction in NF90–NF45 activates the p53 signal pathway in β cells, resulting in inhibition of the β cell expansion under diabetic condition as shown in the islets of bNF45−/− mice fed with HFD (Fig. 3F–H).

## Discussion

Type 2 diabetes (T2D) is a complex and multifactorial disease, which is mainly caused by an insufficient pancreatic β cell response to insulin resistance. Mass of the pancreatic β cell is increased to maintain normoglycemia when insulin resistance occurs, and the dysfunction for cell expansion compensation will progress to T2D^17^. However, the underlying mechanism directing these processes remains largely unknown. Therefore, focusing on the identification of genetic factors involved in β cell proliferation is important to understanding the occurrence of this disease.

In this study, we found that the depletion of NF90–NF45 in murine islet β cells leads to hyperglycaemia owing to a decrement of plasma insulin and less expansion of islet mass under obesity-inducing metabolic stress like high-fat diet (Fig. 3D–H). Intriguingly, the RNA expression of NF45 was significantly reduced in the islets of the T2D patient compared with those of the healthy control (GEO accession no: GSE25724), whereas there is no significant in NF90 expression between the healthy islets and T2D islets (Supplementary Fig. S2). As mentioned above, NF45 and NF90 were known as binding partners that stabilize each other at protein level^13^. Therefore, this observation could support the idea that the downregulation of NF90–NF45 at protein level inhibits β cell expansion in the T2D development. Collectively, these findings suggest that NF90–NF45 is essential for pancreatic β cell function including insulin secretion under the diabetic condition in which an increased insulin resistance is met by morphological and functional compensation of β cells to maintain normoglycemia. Furthermore, the knockdown of NF90–NF45 caused a growth rate retardation in the β cell lines (Fig. 6B) accompanied by an activation of p53 signaling (Figs. 4, 5). Therefore, these findings imply that endogenous NF90–NF45 is essential for islet β cell compensation through silencing of p53 signaling under the diabetic condition.

p53 is a well-known tumor suppressor which enrolled as a key factor in cell cycle arrest, apoptosis and cellular senescence. These activities of p53 are induced by multiple stresses including DNA damage^18^, oxidative stress^19^ and pro-inflammatory cytokines stimulation^20^. Following these stresses, p53 is activated as a transcription factor targeting for genes involved in growth arrest and cell death. p53 also regulates target genes with roles in metabolism, such as the genes GLS2, SCO2, RRAD, and TIGAR^21^. Type 2 diabetes is a metabolic disease characterized by high blood glucose. The first evidence linking p53 to the development of type 2 diabetes was shown by Minamino et al. They demonstrated that inhibition of p53 activity in adipose tissue decreased the expression of proinflammatory cytokines and improved insulin resistance in mice with type 2 diabetes-like disease^22^. Thereafter, Tavana et al. found that p53-mediated senescence of pancreatic β cells develops diabetes in Lig4−/−;p53R172P mice^23^. Lig4−/− mice show non-homologous end-joining (NHEJ) deficiency and embryonic lethality. On the other hand, Lig4−/−; p53−/− mice develop β-cell lymphoma despite of rescue of the embryonic lethal induced by the Lig4 deficiency. A p53 mutant, p53R172P, fails to induce apoptosis but retains the ability of growth arrest and senescence. Importantly, Lig4−/−; p53R172P mice exhibits prevention of the lymphomagenesis, while diabetes occur due to p53-mediated senescence of beta cells in the mice. These findings clearly suggest that the activity of p53 accelerates the development of type 2 diabetes through the senescence of adipose and beta cells. Notably, it is reported that NF90 represses senescence accompanied with elevation of p53 level in fibroblast cells^24^. Further, several studies show that knock-down of NF90(-NF45) leads to a rise in p53 expression in cells^24–28^. We also found the activation of p53 signaling in β cells deleted with NF90–NF45 (Figs. 4, 5). From these findings, taken together, it raises the possibility that the age-related type 2 diabetes are induced by β cell senescence due to the activation of p53 related to a decrease in the NF90–NF45 level.

As shown in Fig. 5, Cdkn1a (also refereed as p21, WAF-1 or CIP1), Pidd1, Zmat3 (also referred as Wig-1), Ccng3 and Casp3 are up-regulated in β cell lines depleted with NF90–NF45 (Fig. 4D). p53 transcribes genes encoding Cdkn1a^15^, Pidd1^29^, Zmat3^30,31^ and Ccng3^32^. On the other hand, the activity of Casp3 is evoked by p53. Cdkn1a, Zmat3 and Ccng3 cause cell cycle arrest, while Pidd1 and Casp3 promote apoptosis. Thus, it is possible that down-regulation of NF90–NF45 in β cells evokes either cell cycle arrest or apoptosis through functions of p53-regulatory factors including Cdkn1a, Pidd1, Zmat3, Ccng3 or Casp3. To address this issue, we performed TUNEL staining to evaluate apoptosis of the islets in the bNF45−/− mice fed with HFD (Supplementary Fig. S3). However, the TUNEL stained signals were undetected in the section from the pancreas containing islets of control and bNF45−/− mice under the HFD feeding although the signals were observed in the sections treated with DNase I which are set as positive controls for staining of the TUNEL (Supplementary Fig. S3). This result suggest that apoptosis would not be occured in primary β cells deleted of NF90–NF45. On the other hand, we observed that the decreased islet mass in the islets of βNF45−/− mice would be engaged in growth retardation in β cells by immunofluorescence with pH3 which is a marker of cell proliferation (Fig. 3I,J). Furthermore, knockdown of NF90–NF45 causes growth retardation of β cell lines (Fig. 6B). In addition, the expression of Cdkn1 and Ccng1, which are known to function as inhibitor of G1 and G2 phase in cell cycle, was significantly elevated in the islets from bNF45−/− mice compared with those from control mice under the HFD feeding (Supplementary Fig. S1). Therefore, the down-regulation of NF90–NF45 would induce growth arrest in primary β cells under obesity-inducing metabolic stress. Taken together, these findings suggest that NF90–NF45 would be positively involved in β cell expansion when the diabetic condition through cell cycle progression of the β cells.

Notably, we found that the down-regulation of NF90–NF45 activates transcription of the luciferase gene controlled by a p53-responsive element^33^ (Fig. 5), suggesting that transcriptional activity of p53 is potentiated by a fall in NF90–NF45 level. As mentioned above, the depletion of NF90(-NF45) causes the elevation of p53 expression in cells^24–28^. Thus, it is likely that NF90–NF45 positively affects quantitative change of p53. On the other hand, qualitative change of p53 is also important for the transcriptional activity of p53. MDM2 acts as a negative regulator for p53 activity through degradation of p53^34^. MDM2 binds to N-terminus of p53 and stimulates the addition of ubiquitin to the C-terminus, which is then degraded^34^. The binding domain of p53 (Ser-15 or Ser-20) to MDM2 becomes phosphorylation following the multiple stress, thereby reducing the binding of MDM2, and resulting in facilitation of p53 transcriptional activity. Furthermore, acetylation on ubiquitination sites of C-terminus of p53 prevents its degradation by ubiquitin proteasome pathway^35^. Therefore, covalent modification (phosphorylation, acetylation and ubiquitination) of p53 N-terminus and C-terminus will be investigated in β cells depleted of NF90–NF45 to uncover the molecular mechanism for activation of p53 transcriptional activity by down-regulation of NF90–NF45.

Consequently, elucidation of quantitative and qualitative changes of p53 by down-regulation of NF90–NF45 in β cells will be of interest but will require extensive work in the future.

## Materials and method

### Animal experiment

All animal experiments were approved by the Committee for Care and Use of Laboratory Animals at Kochi University (approval no. J-0100) and followed the guidelines of ARRIVE. All experiments were performed in accordance with the relevant guidelines and regulations. C57BL/6 mice were used in this study. Organs were removed from sacrificed mice and then flash-frozen by liquid nitrogen or fixed in 10% phosphate-buffered formalin for further analysis. For the high fat diet, mice at 5 weeks of age were randomly assigned into two groups (normal diet or high fat diet groups). Mice were fed with standard-chow or high-fat diet (HFD-60, 60% fat content, Oriental Yeast, Tokyo, Japan) for 22–26 weeks.

### Western blotting

Western blot analysis was performed as described previously^36^. A rabbit anti-ILF3 antibody, mouse anti-Insulin antibody, mouse anti-NF45 antibody and mouse anti-p53 were obtained from Abcam (catalogue no. ab92355, Cambridge, UK), Cell Signaling Technology (#8138, MA, USA) and Santacruz biotechnology (sc-365283 and sc-393031, Texas, USA), respectively. A rabbit anti-NF45 antibody was produced by immunizing New Zealand White rabbits with full-length His-NF45 recombinant proteins, as described previously^37^. Mouse anti-Gapdh (015-25473, Fujifilm Wako, Tokyo, Japan) was used as an internal control. Images were captured and the intensive specific bands were measured using a LAS-4000 mini imaging system (Fujifilm).

### Histological and morphometric analysis

The pancreas was fixed in 10% phosphate-buffered formalin, embedded in paraffin and cut into 3–5 µm thick sections. The tissue slides were de-paraffinized in xylene and rehydrated with ethanol. Immunohistochemistry was performed using a rabbit anti-Ilf3 antibody (ab92355, Abcam), goat anti-Ilf2 antibody (PAB7568, Abnova, Taipei City, Taiwan), mouse anti-Insulin antibody (#8138, Cell Signaling Technology), mouse anti-Glucagon antibody (G2654, Sigma-Aldrich, Merck & Co., Inc. NJ., USA) and mouse anti-phospho-Histone H3 (Ser10) antibody (66863-1-Ig, Proteintech, IL, USA). Fluorescent-conjugated secondary antibodies were purchased from ThermoFisher Scientific, MA, USA. The image was obtained using the fluorescence microscopy instrumentation FV1000D (Olympus, Tokyo, Japan) and all-in-one fluorescence microscope BZ-X800 (Keyence, Osaka, Japan). For morphometric analysis, two individual pancreases were selected from each group. Tissues were embedded into paraffin block. Two to three of tissue sections were collected per depth of 30 mm. Total depth of 120–150 mm was studied. The tissue slides were stained with haematoxylin and eosin (H & E) staining and observed using the light microscope either BZ9000 or BZ-X800 (Keyence, Osaka, Japan). The islet size was examined as the islet area using ImageJ (National Institutes of Health), and the total islet areas were divided by the total number of islets. To quantify phospho-Histone H3 signal in the islet, images of immunofluorescence stain of phospho-Histone H3, counter stain using DAPI and bright field were obtained by BZ-X800. Islet areas and fluorescence intensities were calculated using a hybrid cell count application (BZ-H4C, Keyence, Osaka, Japan) in the BZ-X Analyzer software (BZ-H4A, Keyence, Osaka, Japan). The intensity of phospho-Histone H3 was normalized by the number of DAPI in the islet.

### Islet isolation

Mouse pancreatic islets were isolated as previously described^38^. Briefly, Collagenase XI (C7657, Sigma-Aldrich, MO, USA) was injected through the common bile duct, followed by digestion at 37 °C for 15 min. The islets were separated by Histopaque-1077 using centrifugation at 2400 rpm for 20 min at room temperature.

### Generation of the NF45 flox/flox mice and β-cell-specific NF45-deficient mice

To generate the NF45 flox/flox mice, the CRISPR/Cas9 system was used. To design single guide RNAs (sgRNA), software tools (CHOPCHOP: chopchop.cbu.uib.no/, and Crisprdirect: crispr.dbcls.jp/) were used to predict a unique target site from the murine genome. The sgRNAs were obtained from Integrated DNA Technologies, Inc. (IDT, IA, USA). The lssODNA were prepared using nicking endonucleases as previously reported^39^. Briefly, a plasmid containing two loxP sites flanked by short arms with homology to the NF45 locus was subcloned into pLSODN (BioDynamics Laboratory Inc., Tokyo, Japan) containing the nicking endonucleases binding sequences. 100 µg of purified plasmid DNA was digested with Nb.BsrDI and NotI (New England Biolabs, MA, USA), followed by ethanol precipitation and denatured by the Long ssDNA preparation kit (BioDynamics Laboratory Inc.) according to the manufacturer’s protocol. Single stranded DNA fragments were extracted from the agarose gel and purified with the FAST GeneGel/PCR extraction kit (Nippon Genetic, Tokyo, Japan). For microinjection, 20 ng/µl of Cas9 Nuclease, 20 ng/µl of sgRNA complex and 20 ng/µl of the lssODNA were injected into fertilized eggs from the B6N strain (Japan SLC Inc., Shizuoka, Japan). Mice were identified by PCR analysis using genomic tail DNA as a template. The PCR product amplified from the target region was cloned into the pGEM-T vector (Promega, WI, USA), and the sequence of the PCR product was determined by the Sanger sequence analysis using the purified pGEM-T vector including the PCR product. β cells specific NF45 deficient mice were obtained and maintained by crossing NF45 flox/flox mice with Insulin1-2A-Cre (I2AC) recombinase knock-in mice^14^ purchased from RIKEN BRC, Tsukuba, Japan.

### Measurement of blood glucose and plasma insulin level

Blood glucose in murine tail vein was measured using a blood glucose meter (TERUMO, Tokyo, Japan). For the measurement of the murine plasma insulin level, blood was collected from the murine tail vein. The serum was obtained by centrifugation and insulin in the serum was measured by ELISA (Morinaga Institute of Biological Science, Kanagawa, Japan).

### Cell culture

For the murine insulinoma cell lines, Beta-TC-6 (BTC6) cells were purchased from ATCC® (VA, USA). The cells were cultured in Dulbecco’s Modified Eagle’s Medium (DMEM) supplemented with 25 mM glucose, 15% heat-inactivated foetal bovine serum (FBS) and 1% penicillin–streptomycin.

### RNA interference

BTC6 cells were transfected with a mixture of three stealth RNAi duplexes (Invitrogen, MA, USA) that targeted NF90 and NF45, and the stealth RNAi negative control (Invitrogen) was used as a negative control. Lipofectamin RNAiMAX reagent (Invitrogen) was used for transfection. For the treatment of siRNAs for 7 days, the cells were detached and reseeded to a new dish after cells with siRNAs were cultured for 3 days. Transfection with siRNAs was then performed the next day using the seeding cells, and the cells were cultured for another 3 days.

### Whole-genome expression microarray

Microarray analysis was performed as described previously^10^. Briefly, the total RNA was isolated from BTC6 cells treated with siRNAs for 7 days. Single-stranded cDNA was generated from the amplified cRNA derived from the total RNA and labelled biotin with the GeneChip® WT PLUS Reagent Kit (Affymetrix, MA, USA). Following fragmentation by the GeneChip® WT PLUS Reagent Kit (Affymetrix), the single-stranded cDNA was hybridized with a MoGene2.1ST array strip (Affymetrix). The array was scanned using the GeneAtlas system (Affymetrix) to measure the intensities of the microarray spots. The intensities of the spots were analysed by the Transcriptome Analysis Console (TAC) Software (ThermoFisher). All microarray data have been deposited in the Gene Expression Omnibus (GEO) database, accession no. GSE184835.

### qRT-PCR

Total RNA was isolated from BTC6 cells by Sepazol (Nacalai Tesque, Kyoto, Japan). cDNA was synthesized from the RNA using SuperScript III reverse transcriptase (Invitrogen) with a random hexamer primer according to the manufacture’s protocol (Invitrogen). Quantitative Real-time PCR was performed by StepOne PLUS (Applied Biosystems, MA, USA) using the SYBR Green PCR Master Mix (Applied Biosystems).

### Reporter assay

Cells were co-transfected with either siNTC or siNF90–NF45 together with reporter plasmids using Lipofectamine 2000 according to the manufacturer’s instructions (Thermo Fisher Scientific). After 72 h, cells were lysed by passive lysis buffer (Promega) and the luciferase activity was measured with the Dual-Luciferase Reporter Assay System (Promega) using the GLOMAX 20/20 Luminometer (Promega).

### Plasmids

PG13-luc (wt p53 binding sites) and MG15-luc (mut p53 binding sites) were a gift from Dr. Bert Vogelstein (Addgene plasmid #16442 and #16443, respectively)^15^. pCE-mp53DD was a gift from Dr. Shinya Yamanaka (Addgene plasmid #41856)^16^.

### Cell proliferation assay

Cell viability following 0–5 days of culture was examined by 3-(4,5-dimetylthiazol-2-yl)-5-(3-carboxymethoxyphenyl)-2-(4-sulfophenyl)-2H-tetrazolium (MTS) assay. Three days posttransfection, siRNA together with or without an expression plasmid-transfected cells were 20,000 cells/well in 96-well plate. Viability of the cells following 0, 1, 3 and 5 days of culture was assayed by staining the MTS reagent (CellTiter 96 AQueous One Solution Cell Proliferation Assay, Promega) and measuring the optical density at 490 nm by SpectraMax 190 microplate Reader (Molecular Devices).

Sequences of all oligonucleotides are listed in Supplementary Table S1.

## Supplementary Information

Supplementary Figure 1.

Supplementary Figure 2.

Supplementary Figure 3.

Supplementary Figure 4.

Supplementary Figure 5.

Supplementary Figure 6.

Supplementary Figure 7.

Supplementary Table S1.
