# Supplementary figures and images for: NF90–NF45 is essential for β cell compensation under obesity-inducing metabolic stress through suppression of p53 signaling pathway
Source: Sci Rep. 2022 May 25;12:8837. doi: 10.1038/s41598-022-12600-y (PMC9132887; doi:10.1038/s41598-022-12600-y)

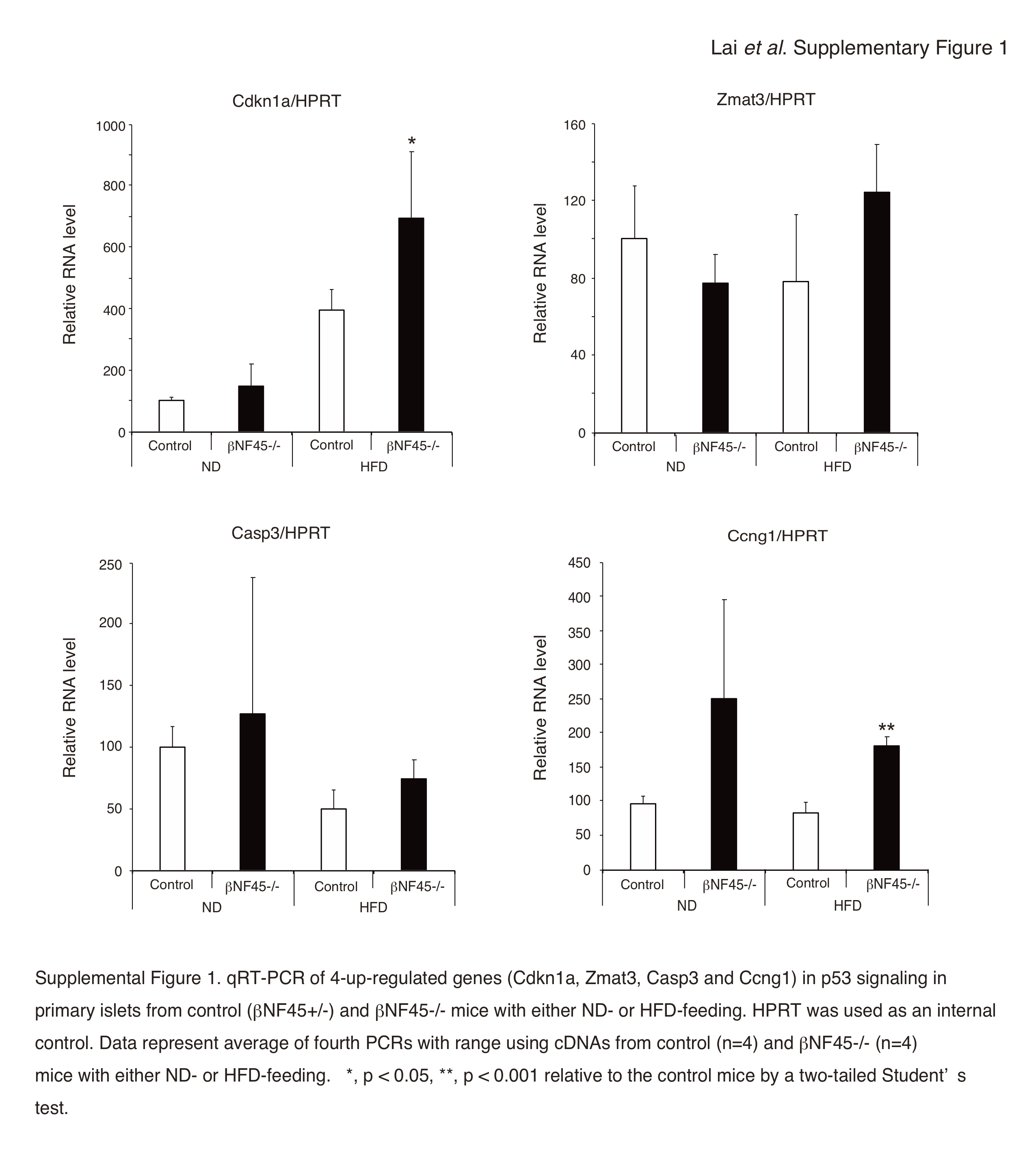

Supplement: Supplementary file 1 — Supplementary Figure 1. [file 41598_2022_12600_MOESM1_ESM.jpg]

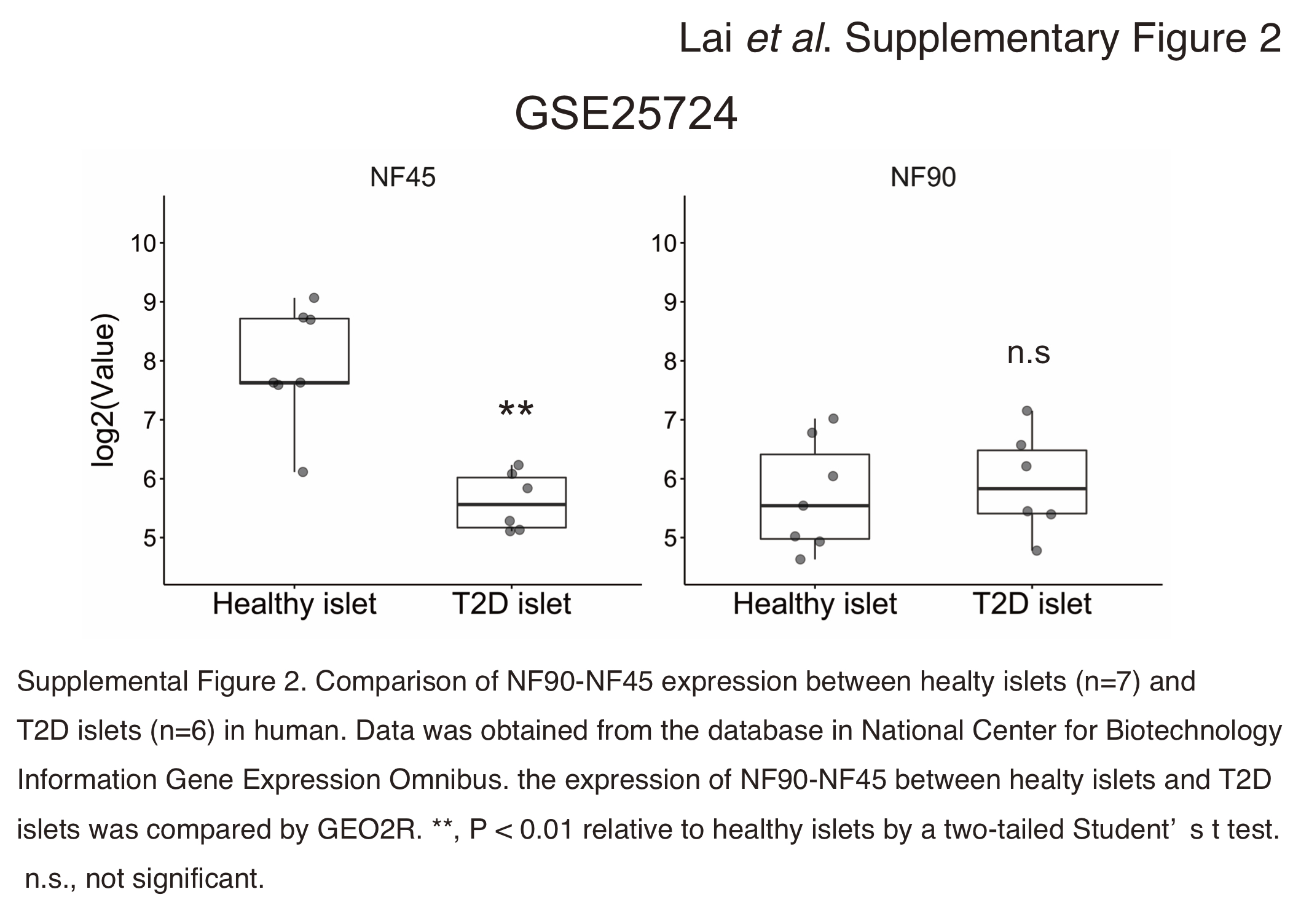

Supplement: Supplementary file 2 — Supplementary Figure 2. [file 41598_2022_12600_MOESM2_ESM.jpg]

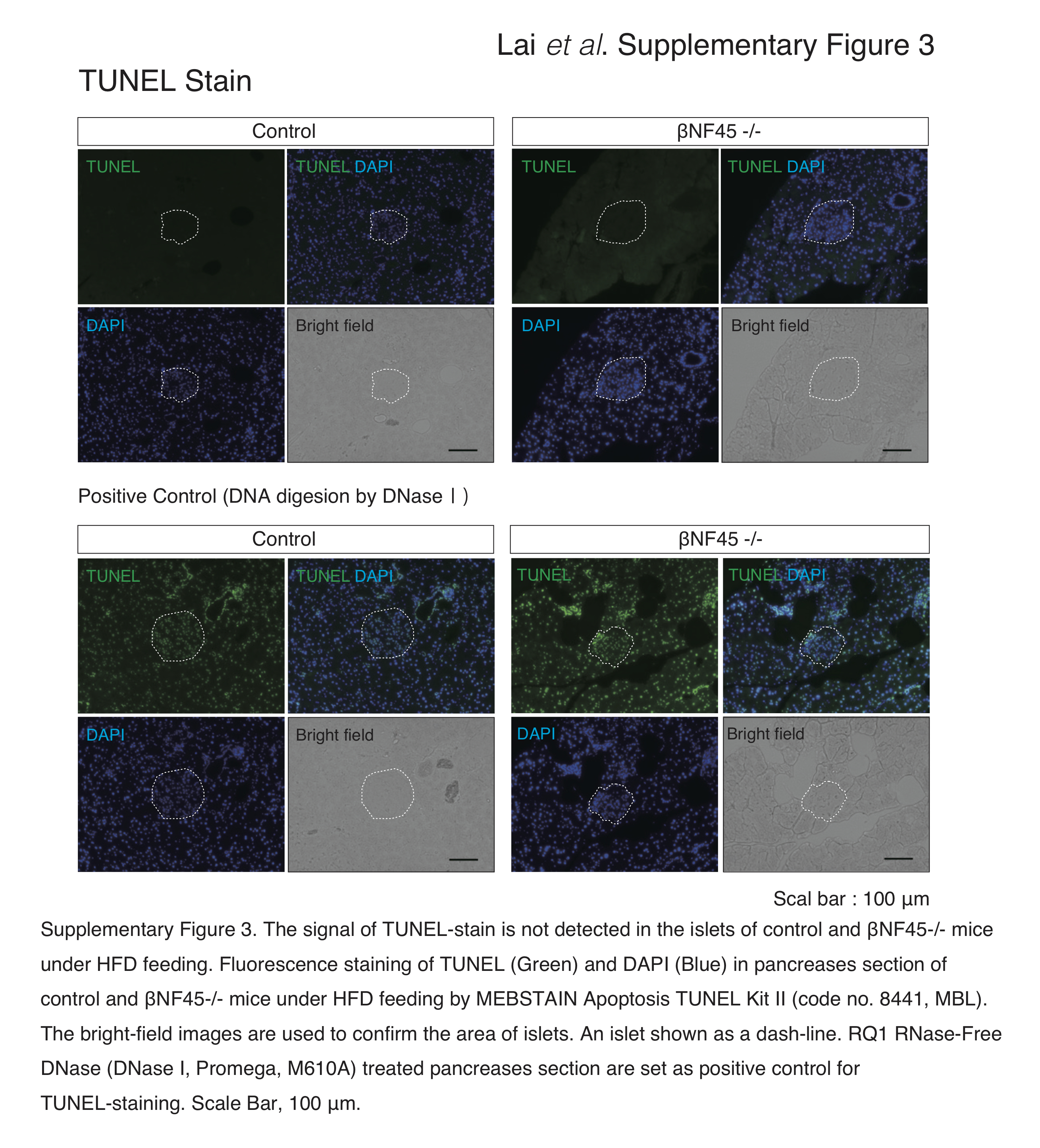

Supplement: Supplementary file 3 — Supplementary Figure 3. [file 41598_2022_12600_MOESM3_ESM.jpg]

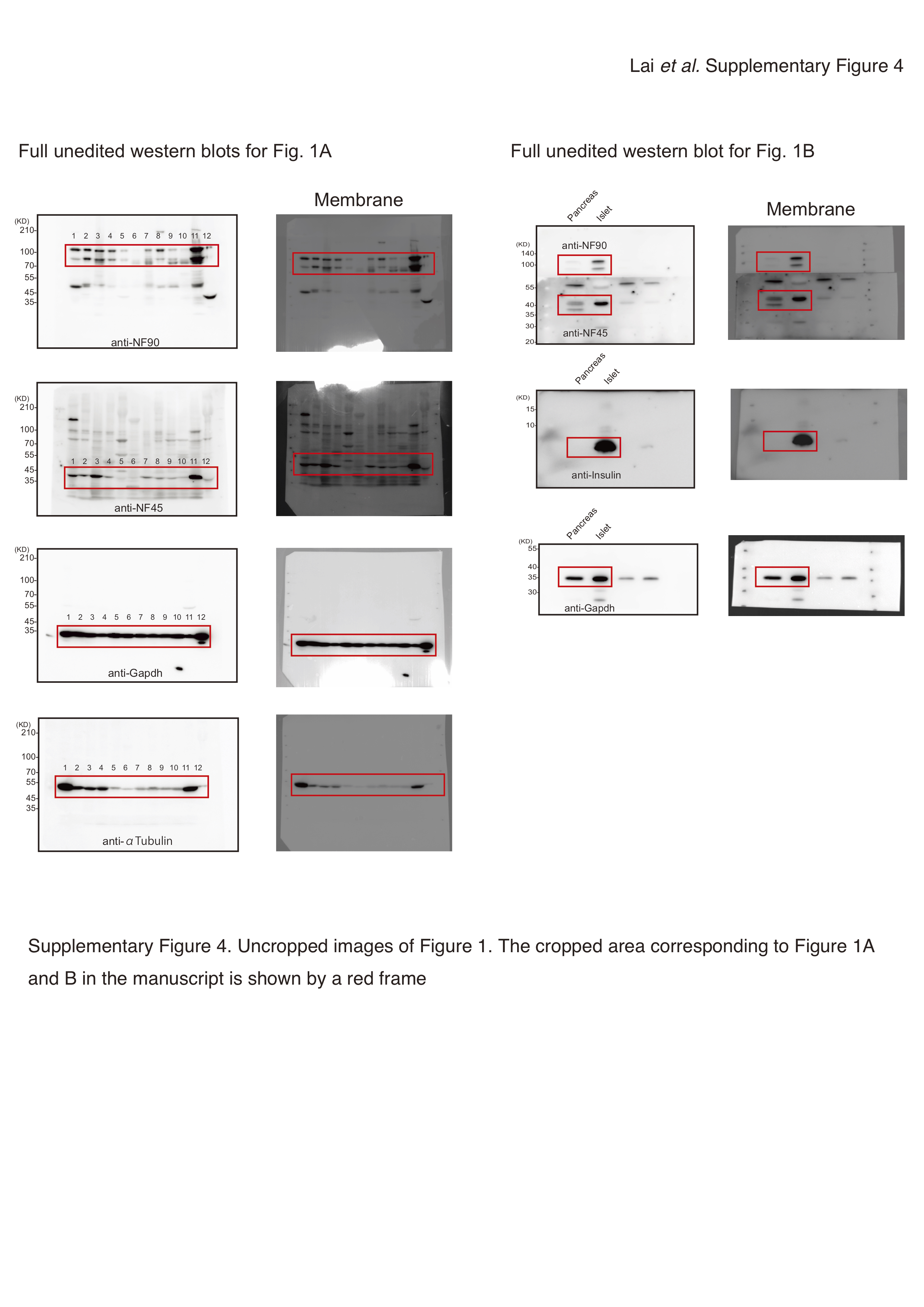

Supplement: Supplementary file 4 — Supplementary Figure 4. [file 41598_2022_12600_MOESM4_ESM.jpg]

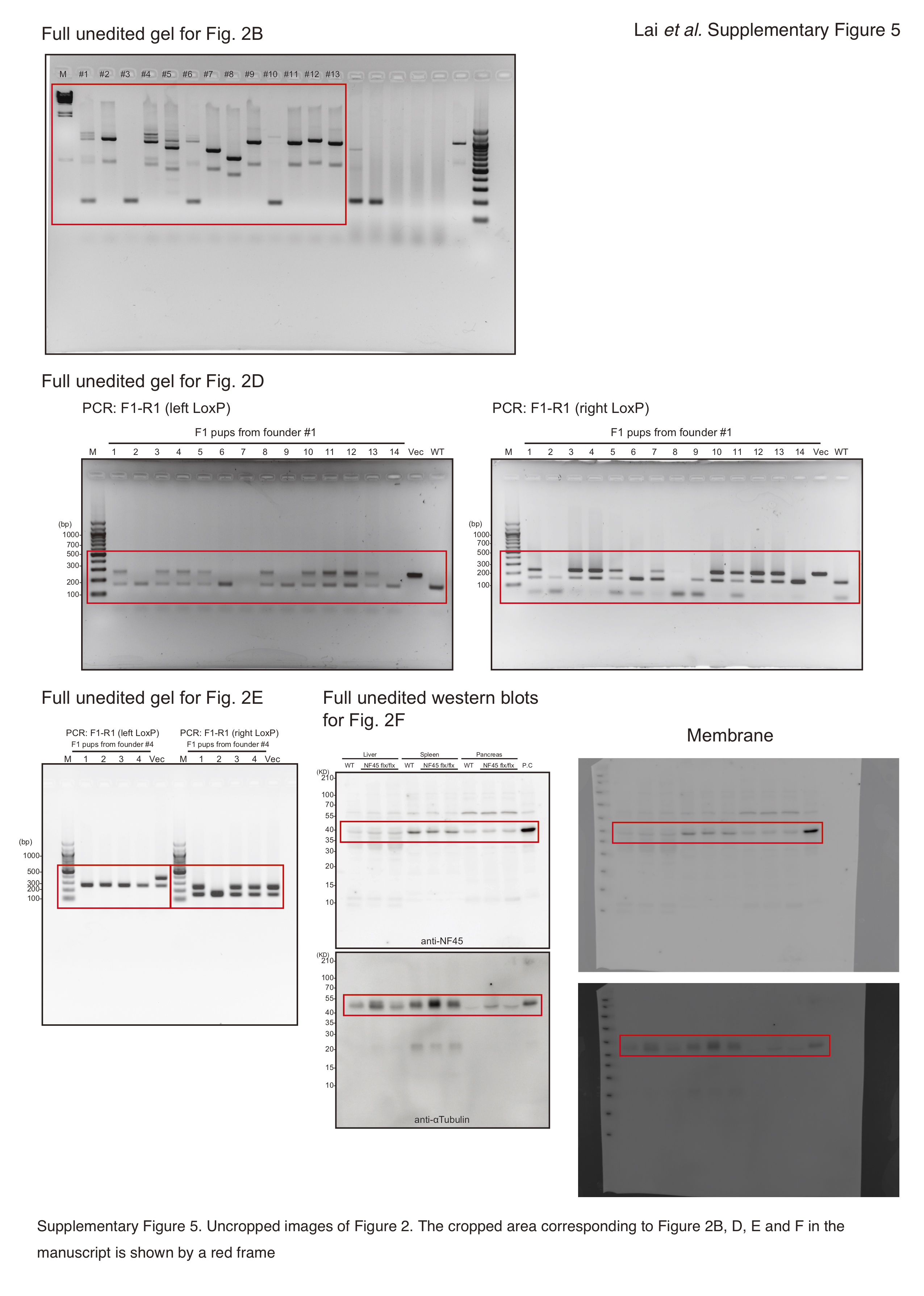

Supplement: Supplementary file 5 — Supplementary Figure 5. [file 41598_2022_12600_MOESM5_ESM.jpg]

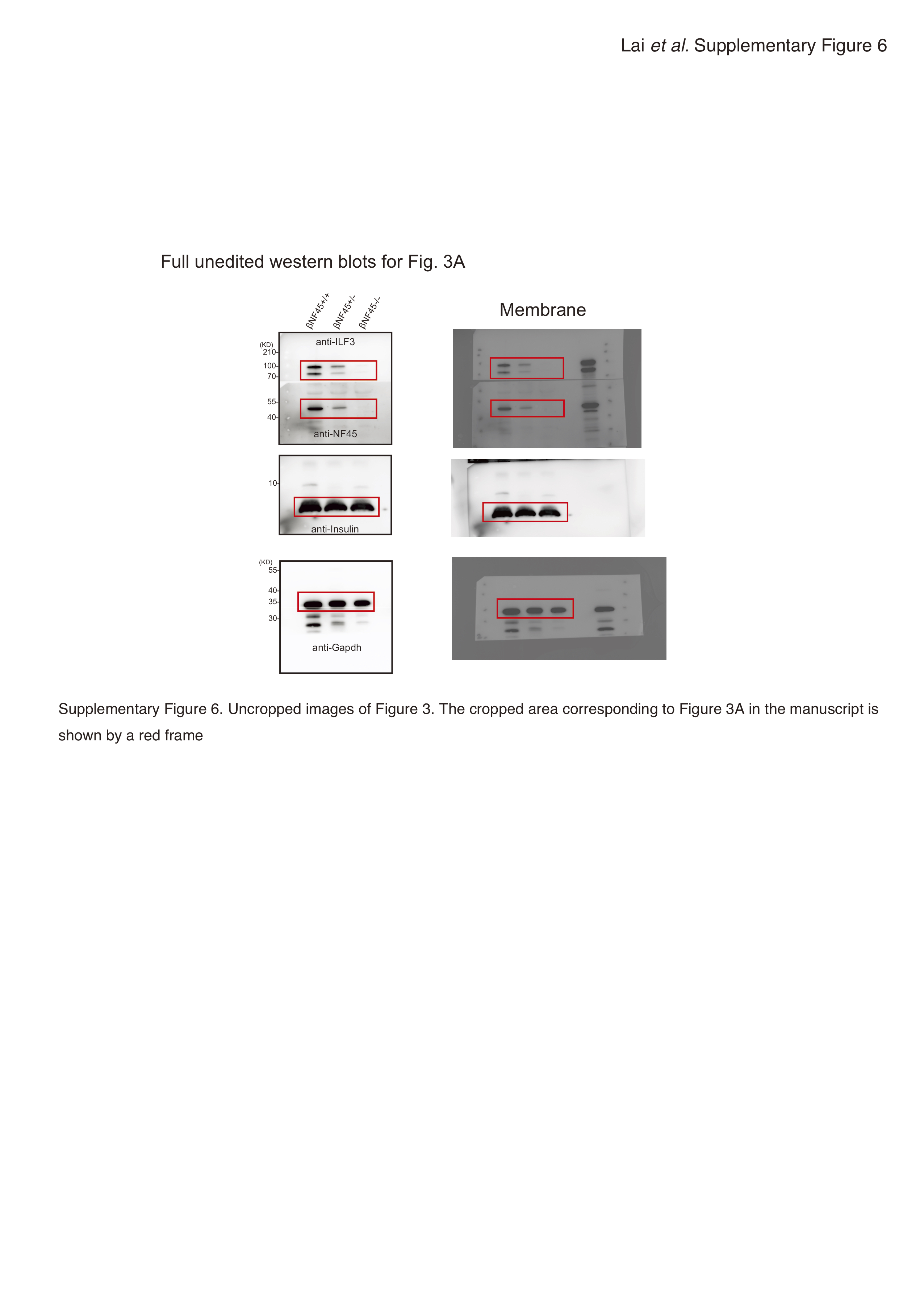

Supplement: Supplementary file 6 — Supplementary Figure 6. [file 41598_2022_12600_MOESM6_ESM.jpg]

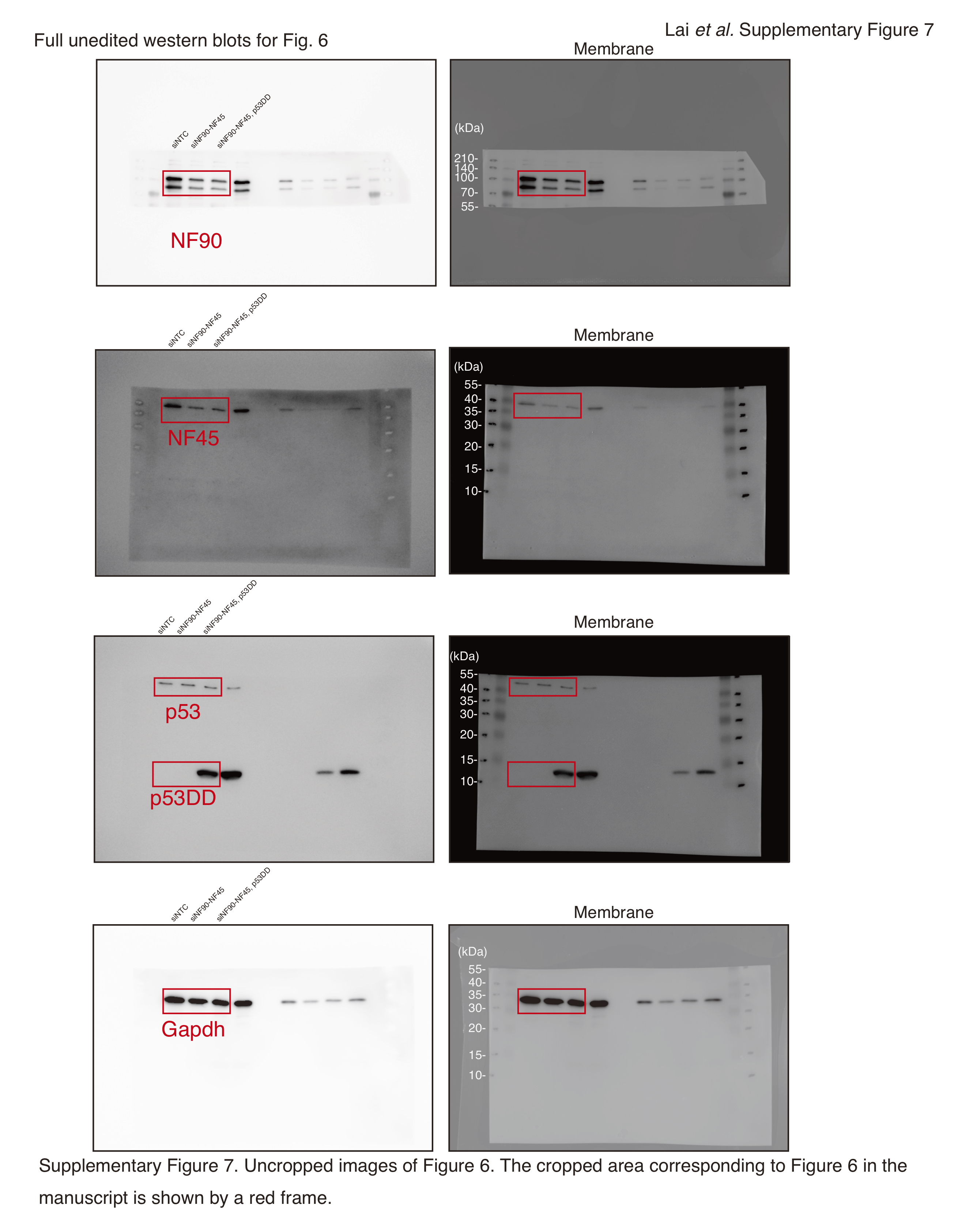

Supplement: Supplementary file 7 — Supplementary Figure 7. [file 41598_2022_12600_MOESM7_ESM.jpg]
